# A new molecular prognostic score for predicting the risk of distant metastasis in patients with HR+/HER2− early breast cancer

**DOI:** 10.1038/srep45554

**Published:** 2017-03-28

**Authors:** Gyungyub Gong, Mi Jeong Kwon, Jinil Han, Hee Jin Lee, Se Kyung Lee, Jeong Eon Lee, Seon-Heui Lee, Sarah Park, Jong-Sun Choi, Soo Youn Cho, Sei Hyun Ahn, Jong Won Lee, Sang Rae Cho, Youngho Moon, Byung-Ho Nam, Seok Jin Nam, Yoon-La Choi, Young Kee Shin

**Affiliations:** 1 Department of Pathology, University of Ulsan College of Medicine, Asan Medical Center, Seoul, 05505, Korea; 2 College of Pharmacy, Kyungpook National University, Daegu, 41566, Korea; 3 Research Institute of Pharmaceutical Sciences, College of Pharmacy, Kyungpook National University, Daegu, 41566, Korea; 4 Gencurix, Inc., Seoul, 08394, Korea; 5 Department of Surgery, Samsung Medical Center, Sungkyunkwan University School of Medicine, Seoul, 06356, Korea; 6 Department of Nursing Science, College of Nursing, Gachon University, Incheon, 21936, Korea; 7 The Center for Anti-cancer Companion Diagnostics, Bio-MAX/N-Bio, Seoul National University, Seoul, 08826, Korea; 8 Department of Pathology and Translational Genomics, Samsung Medical Center, Sungkyunkwan University School of Medicine, Seoul, 06356, Korea; 9 Department of Surgery, University of Ulsan College of Medicine, Asan Medical Center, Seoul, 05505, Korea; 10 Department of Cancer Control and Policy, Graduate School of Cancer Science and Policy, National Cancer Center, Goyang, Gyeonggi-do, 10408, Korea; 11 Laboratory of Cancer Genomics and Molecular Pathology, Samsung Biomedical Research Institute, Samsung Medical Center, Sungkyunkwan University School of Medicine, Seoul, 06356, Korea; 12 Department of Health Sciences and Technology, SAIHST, Sungkyunkwan University, Seoul, 06356, Korea; 13 Department of Molecular Medicine and Biopharmaceutical Sciences, Graduate School of Convergence Science and Technology, Seoul National University, Seoul, 08826, Korea; 14 Laboratory of Molecular Pathology and Cancer Genomics, College of Pharmacy, Seoul National University, Seoul, 08826, Korea

## Abstract

To make an optimal treatment decision for early stage breast cancer, it is important to identify risk of recurrence. Here, we developed and validated a new prognostic model for predicting the risk of distant metastasis in patients with pN0-N1, hormone receptor-positive, HER2-negative (HR+/HER2−) breast cancer treated with hormone therapy alone. RNA was extracted from formalin-fixed, paraffin-embedded tumor tissues and gene expression was measured by quantitative real-time reverse transcription-PCR. The relative expression of six novel prognostic genes was combined with two clinical variables (nodal status and tumor size) to calculate a risk score (BCT score). In the validation cohort treated with hormone therapy alone, the 10 year rate of distant metastasis in the high-risk group (26.3%) according to BCT score was significantly higher than that in the low-risk group (3.8%) (*P* < 0.001). Multivariate analysis adjusted for clinical variables revealed that BCT score is an independent predictor of distant metastasis. Moreover, the C-index estimate revealed that BCT score has a prognostic power superior to that of prognostic models based on clinicopathological parameters. The BCT score outperforms prognostic models based on traditional clinicopathological factors and predicts the risk of distant metastasis in patients with HR+/HER2− early breast cancer.

Hormone receptor (HR)-positive breast cancer, including HR-positive, human epidermal growth factor receptor 2 (HER2)-negative (HR+/HER2−) and HR-positive, HER2-positive (HR+/HER2+) breast cancer, accounts for a large proportion of breast cancer cases; indeed, HR+/HER2− breast cancer is the most common subtype^1^,^2^. Patients with HR+/HER2− early breast cancer have a higher risk of late recurrence beyond 5 years after primary hormone therapy than those with HR+/HER2+ or HR- breast cancer^3^,^4^,^5^,^6^. For this population, accurate assessment of risk of recurrence beyond 5 years would be useful for decision-making in terms of whether to extend adjuvant hormone therapy or treat with adjuvant chemotherapy. Clinicopathological parameters such as tumor size and lymph node (LN) status have been used as traditional prognostic factors and several prognostic models (e.g., Nottingham Prognostic Index [NPI]^7^,^8^, SNAP^9^ and PREDICT^10^,^11^) are now available to estimate the survival of breast cancer patients. These models all are based on known clinical prognostic factors but the clinical variables for each model are slightly different. The models also showed different prognostic performance^11^. However, current clinicopathological parameters alone and prognostic models based on clinical variables have limited predictive or prognostic value for recurrence risk in patients with early breast cancer. Accordingly, there have been numerous attempts to identify clinically significant prognostic factors beyond standard clinical variables, which may be associated with disease recurrence or distant metastasis in HR+ early breast cancer patients.

Gene expression-based approaches provide significant prognostic or predictive information with respect to breast cancer. Several commercial assays based on expression of multiple genes in frozen or formalin-fixed, paraffin-embedded (FFPE) samples have been developed^12^,^13^,^14^,^15^,^16^,^17^,^18^. The recurrence score (RS) from the Oncotype DX assay^19^ and the risk of recurrence score from Prosigna (based on the PAM50 gene signature)^20^ are used to predict the risk of distant recurrence after hormone therapy. These assays are useful for discriminating high- and low-risk patients with estrogen receptor-positive (ER+) early breast cancer as they provide more information than clinical variables alone. The multigene risk score provided by the recently developed EndoPredict test (EP) also provides additional prognostic information about the risk of distant recurrence of ER+/HER2− breast cancer independent of traditional clinicopathological parameters^18^. In addition to multigene expression-based scores, an immunohistochemical score (IHC4 score) based on expression of four markers (ER, progesterone receptor (PR), Ki-67, and HER2) provides prognostic information similar to that derived from the RS^21^.

However, these tests produce discordant results when used for risk assignment^22^, and studies show that the results of the 70-gene prognostic signature MammaPrint are different in Asian breast cancer patients and patients from Europe^23^,^24^. This suggests that the performance of the current commercial prognostic assays is population-specific. Furthermore, currently available assays that use multigene expression signatures mainly based on proliferation-related genes have certain limitations. Assays such as Oncotype DX or MammaPrint target ER+ breast cancer but do not discriminate between ER+/HER2− and ER+/HER2+ subtypes. Although both are subtypes of ER+ breast cancer, they have different prognoses after hormone therapy and show different responses to adjuvant chemotherapy^25^. HR+/HER2− breast cancer is generally associated with a lower risk of recurrence after hormone therapy than HR+/HER2+ breast cancer^2^,^25^, whereas more than half of distant recurrences in patients with LN−, HR+/HER2− breast cancer occur after 5 years^4^,^20^. Because adjuvant chemotherapy is of little benefit to patients at low risk of recurrence, this treatment is generally used for HR+/HER2− breast cancer patients considered at high risk of recurrence or for those with an unfavorable prognosis due to low HR expression and high cell proliferation^26^,^27^. By contrast, current treatment guidelines based on tumor size and nodal status recommend appropriate adjuvant chemotherapy in addition to hormone therapy and anti-HER2 therapy for many HR+/HER2+ cases. In this context, it is of particular importance to assess the benefit of chemotherapy for patients with HR+/HER2− breast cancer. However, currently available assays such as Oncotype DX are optimized to identify high-risk patients among ER+ early breast cancer cases and do not differentiate those with HR+/HER2− breast cancer. Therefore, there is an urgent clinical need to identify novel prognostic markers to better differentiate high- and low-risk patients with HR+/HER2− early stage breast cancer.

In a previous study, we used public microarray gene expression data to identify 384 candidate prognostic genes, all of which are associated with distant metastasis in patients with LN− early breast cancer^28^. From these 384 genes, we selected 16 candidate prognostic genes and examined the association between their expression levels by a quantitative real-time reverse transcription-PCR (qRT-PCR) assay and clinical outcome in a large number of breast cancer FFPE samples.

Finally, we selected the six genes to develop a prognostic model to predict the risk of distant recurrence or distant metastasis in HR+/HER2− early breast cancer. Here, we describe the development of a new molecular predictor of distant metastasis, referred to as the BCT score, for patients with pathologic N0 or N1 status (pN0-N1), HR+/HER2− breast cancer treated with hormone therapy alone. This score was based on a combination of six prognostic genes and two clinical variables and was validated in an independent cohort. Furthermore, we assessed its ability to predict the risk of distant metastasis in HR+/HER2− early breast cancer by comparing its performance with that of conventional clinicopathological risk factors.

## Results

### New prognostic algorithm for predicting the risk of distant metastasis in those with HR+/HER2− early breast cancer

Of the 906 patients with pN0-N1, HR+/HER2− breast cancer included in the study, 174 (19.2%) who were treated with hormone therapy alone were used to develop the BCT score. The detailed clinical characteristics of all patients included in this discovery cohort are shown in Table 1. The median age was 53.8 (24.3–80.5) years, and most patients had pN0 tumors (93.7%). The majority of tumors were small (≤ 2 cm; 81.0%). Patients with pathologic stages IA–IIB were included; 78.2% were stage IA.

**Figure 1:**
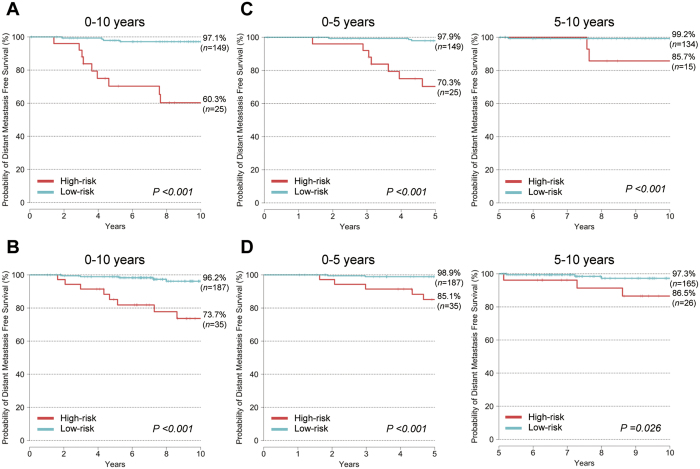
Kaplan-Meier plots of distant metastasis in high- and low-risk groups (as defined by the BCT score) in the discovery cohort and validation cohort. Kaplan-Meier plots of distant metastasis at 10 years, between 0–5 years, and at 5–10 years in patients from the discovery cohort (**A**,**C**) and validation cohort (**B**,**D**). The cutoff value for the BCT score was 4.

The BCT score in the discovery cohort ranged from 0.00–7.12 (median, 2.47) (data not shown). Patients in the discovery cohort were classified into low-risk and high-risk groups according to a pre-specified cutoff BCT score of 4, as described in the Method section. Accordingly, 85.6% (*n* = 149) of breast cancer patients were assigned to the low-risk group, whereas 14.4% (*n* = 25) were deemed to be at high risk of distant metastasis (Fig. 1A). The Kaplan-Meier survival curve showed a statistically significant difference in distant metastasis-free survival (DMFS) between the low-risk and high-risk groups (*P* < 0.001). Probability estimates of 10 year DMFS for patients in the low-risk and high-risk groups were 97.1% and 60.3%, respectively (Fig. 1A). In other words, distant metastasis rates at 10 years in patients assigned to the low-risk and high-risk groups were 2.9% and 39.7%, respectively. This result demonstrates that the BCT score clearly differentiates patients at high and low risk of distant metastasis. In subgroup analyses, a higher BCT score was associated with significantly increased risk of distant metastasis in all analyzed subgroups including age, tumor size, histologic grade, pathologic stage and pN status, except for those with pN1 tumors (Supplementary Table S1). We also found that these significant differences in distant metastasis between low-risk and high-risk patients are consistent throughout all subgroups, except for those with pN1 tumors (Supplementary Fig. S1).

We then examined the association between the BCT score and distant metastasis using Cox’s proportional hazard model. A multivariate analysis in which the BCT score was evaluated in relation to other clinical variables or prognostic models based on clinicopathological factors revealed that the BCT score correlated significantly with distant metastasis (*P* = 0.001), whereas models based on clinical variables or other prognostic factors did not (Tables 2 and 3). This shows that the BCT score is an independent negative prognostic factor for distant metastasis in patients with pN0-N1, HR+/HER2− breast cancer.

### Validation of the BCT score in an independent cohort

The BCT score was independently validated in 222 patients. As in the discovery cohort, all patients had HR+/HER2− early breast cancer (pN0-N1) and were treated with hormone therapy alone (Table 1). Apart from age, menopausal status, and histologic grade, the clinical characteristics of the validation cohort were similar to those of the discovery cohort. The validation cohort contained a greater number of younger patients, premenopausal patients, and patients with higher histologic grade. The association between the BCT score and distant metastasis was assessed using Cox models adjusted for clinical variables including age, tumor size, number of LN metastases, histologic grade, ER and PR levels by immunohistochemistry (IHC). Multivariate analysis revealed that the BCT score was independently associated with distant metastasis (hazard ratio, 1.59; 95% confidence interval [CI], 1.12–2.25; *P* = 0.009), whereas clinical variables were not significant (Table 2). By contrast, other prognostic models based on clinicopathological parameters alone (the NPI score, PREDICT, and SNAP) were not independent predictors of distant metastasis (Table 3).

Patients were divided into low-risk and high-risk groups according to the cutoff value for the BCT score pre-specified in the discovery cohort. The distribution of the BCT score in the validation cohort was similar to that in the discovery cohort (data not shown). Compared with the discovery cohort, more patients (15.8%; 35/222) in the validation cohort were classified as high-risk according to the BCT score, whereas 187 patients were classified as low-risk. Kaplan-Meier survival analyses revealed significant differences in distant metastasis rates between low-risk and high-risk patients according to the BCT score (*P* < 0.001) (Fig. 1B). For example, the 10 year distant metastasis rates for patients in the low-risk and high-risk groups were 3.8% and 26.3%, respectively.

We next examined the prognostic value of the BCT score in subgroups of patients in the validation cohort who were treated with hormone therapy alone (*n* = 222). A higher BCT score was associated with a significantly increased risk of distant metastasis in those aged ≥50 years, those with histologic grade 2 and pN0 tumors (Supplementary Table S1). Kaplan-Meier analyses showed significant differences in distant metastasis between low-risk and high-risk patients with tumor size ≤2 cm and >2 cm, those with histologic grade 2 and 3, those with pathologic stage 1 and 2 as well as those aged ≥50 years and those with pN0 tumors (Supplementary Fig. S2). These results indicate that the BCT score is useful for identifying high risk patients among these subgroups.

### Ability of the BCT score to predict late distant metastasis

In the discovery cohort, the BCT score suggested significant differences between the low-risk and high-risk groups in terms of both early (0–5 years) and late metastasis (5–10 years) (*P* < 0.001) (Fig. 1C). Therefore, we examined the ability of the BCT score to predict early and late metastases in the validation cohort. Kaplan-Meier analysis revealed that the low-risk group had a significantly higher probability of DMFS than the high-risk group at both 0–5 years (*P* < 0.001) and 5–10 years (*P* = 0.026) (Fig. 1D). The low-risk group showed a probability of DMFS of 98.9% (97.4–100.0%) within 5 years and 97.3% (94.2–100.0%) at between 5 and 10 years. These results show that the BCT score has prognostic value for predicting late distant metastasis as well as early distant metastasis.

### Comparing the prognostic performance of the BCT score with that of other clinicopathological risk factors

**Figure 2:**
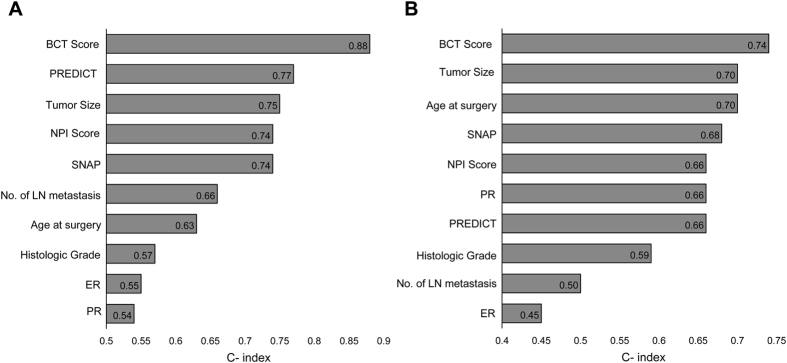
The prognostic performance of the BCT score compared with that of clinical variables or other prognostic models according to the C-index. (**A**) Discovery cohort. (**B**) Validation cohort. C-index estimates for clinical variables (age at the time of surgery, tumor size, number of LN metastases, histologic grade, ER and PR levels by IHC), the prognostic models (NPI score, PREDICT, and SNAP), and the BCT score are shown. ER: estrogen receptor, IHC: immunohistochemistry, LN: lymph node, PR: progesterone receptor.

We next compared the prognostic performance of the BCT score with other clinical variables or clinical prognostic models in terms of its ability to predict the risk of distant metastasis at 10 years in those with pN0-N1, HR+/HER2− breast cancer. First, we calculated the Harrell’s concordance index (C-index) for the BCT score, various clinical variables (age at surgery, tumor size, number of LN metastases, histologic grade, ER and PR levels by IHC), and the established clinical prognostic models (the NPI score, PREDICT, and SNAP) in the validation cohort. As shown in Fig. 2, the BCT score with the highest C-index (0.88) in the discovery cohort was also the best predictor of the risk of distant metastasis (highest C-index, 0.74) in the validation cohort. This finding demonstrates that the BCT score provides more prognostic information about the risk of distant metastasis than established prognostic models based on clinical variables. These results indicate that, in early breast cancer patients, the BCT score is a more powerful predictor for distant metastasis than clinical variables.

### Performance of the BCT score in chemotherapy-treated patients

Although the BCT score was developed to predict the risk of distant metastasis in patients with pN0-N1, HR+/HER2− breast cancer treated with hormone therapy alone, we decided to examine its ability to predict the risk of distant metastasis in patients treated with hormone therapy plus chemotherapy. Five hundred and ten patients were included in the analysis. In the validation cohort, there were significant differences between patients treated with hormone therapy alone (*n* = 222) and chemotherapy-treated patients (*n* = 510) in terms of age, pN, tumor size, and pathologic stage (*P* < 0.001) (Table 1). Compared with patients treated with hormone therapy alone, patients treated with chemotherapy were younger, were pN1, had larger tumors, and were at an advanced pathologic stage. That is, the clinicopathological characteristics of chemotherapy-treated patients were different from those of patients treated with hormone therapy alone. The 510 patients were classified as high-risk (*n* = 267) or low-risk (*n* = 243) according to the BCT score. We found a significant difference in 10 year distant metastasis rates between the high-risk (21.8%) and low-risk (9.3%) groups (*P* < 0.001) (Supplementary Fig. S3). Subgroup analysis of chemotherapy-treated patients revealed that a higher BCT score was associated with a significantly increased risk of distant metastasis in all subgroups, except for those with histologic grade 3 and pathologic stage I tumors (Supplementary Table S1). These findings demonstrate that the BCT score can discriminate patients at high risk and low risk of distant metastasis after chemotherapy treatment.

## Discussion

Here, we developed a new molecular prognostic signature (called the BCT score) to predict the risk of distant metastasis in patients with pN0-N1, HR+/HER2− breast cancer treated with hormone therapy alone. The score was based on a combination of qRT-PCR gene expression data for six prognostic genes and two clinical variables. The prognostic value of the BCT score was validated in independent cohorts.

Several multigene assays have been developed to predict the risk of recurrence in patients with pN0-N1, ER+ breast cancer; these assays should be more precise than forecasts based on traditional clinical prognostic factors or the status of HRs (ER or PR) and/or HER2^12^,^15^,^16^,^18^. Nevertheless, the status or expression of ER/PR or HER2-related genes is still an important factor utilized by these multigene assays. For example, the 21-gene Oncotype DX assay includes the estrogen-related (*ESR1, PGR, BCL2*, and *SCUBE2*) and HER2-related (*GRB7, HER2*) groups of genes^12^. In addition, each molecular intrinsic subtype is significantly related to the status or expression level of HRs and HER2 in the 50-gene PAM50 assay^16^. The recently developed EP score also includes several ER-associated genes: *AZGP1* and *RBBP8* (correlated with ER), *IL6ST* and *STC2* (co-regulated by ER), and *MGP* (induced by ER)^18^. On the other hand, expression of the six prognostic genes used herein did not show a high correlation with that of ER, PR, and/or HER2 (data not shown).

Furthermore, molecular characterization of breast cancer subgroups identified subtype-specific gene signatures^16^,^29^,^30^,^31^,^32^, and gene signatures associated with prognosis differ between the subtypes^33^. Major characteristic expression signatures associated with ER+ breast cancer prognosis are related to expression of cell proliferation-related genes^33^; accordingly, current commercial multigene assays for ER+ breast cancer mainly comprise proliferation-related genes. Notably, the BCT algorithm is a prognostic model that encompasses two major biological processes, cell proliferation and the immune response, both of which are significantly related to the clinical outcome of patients with LN− breast cancer^28^. In our previous study, we found that higher expression of five proliferation-related genes (*UBE2C, TOP2A, RRM2, FOXM1*, and *MKI67*) was associated with shorter DMFS in patients with LN−, HR+/HER2− breast cancer, whereas expression of the immune response-related *BTN3A2* gene was positively correlated with longer DMFS. An association between expression of proliferation-related genes included in the BCT algorithm and prognosis of breast cancer patients has been reported previously^34^,^35^,^36^. Our findings highlight the importance of utilizing expression of immune response-related genes in addition to expression of proliferation-related genes as valuable prognostic factors for distant metastasis in patients with pN0-N1, HR+/HER2− breast cancer. The immune response signature is associated with the prognosis of ER−/HER2− and ER−/HER2+^33^ but not with that of ER+ breast cancer. In this context, it is of critical importance that our prognostic model for the risk of distant metastasis in HR+ breast cancer includes expression of *BTN3A2*. It is also notable that our model relies on expression levels of a relatively small number of genes when compared with the number detected by other multigene assays.

*BTN3A2* encodes a member of the immunoglobulin superfamily and is involved in the T cell-mediated immune responses; as such, it is considered a possible factor associated with favorable prognosis in ovarian cancer patients^37^,^38^. However, the prognostic value of expression of this gene in breast cancer is unclear. Here, for the first time, we show that combining expression of *BTN3A2* with that of proliferation-related genes allows reliable prediction of the risk of distant metastasis. Furthermore, *BTN3A2* expression is itself associated with favorable prognosis in pN0-N1, HR+/HER2− breast cancer.

The validation study demonstrated the prognostic value of the BCT score for predicting 10 year distant metastasis in early breast cancer patients treated with hormone therapy alone. According to the BCT score, the rate for 10 year distant metastasis in high-risk patients was 26.3%, whereas that in low-risk patients was 3.8%. This clearly shows that the BCT score reliably identified patients likely to have a good clinical outcome and who therefore may not require extended hormone therapy or additional adjuvant chemotherapy. In addition, multivariate analysis revealed that the BCT score was an independent predictor of distant metastasis, whereas prognostic models based on traditional clinicopathological parameters, such as NPI score, PREDICT, and SNAP, did not retain significance. Furthermore, we found that the BCT score had a higher C-index value than other clinical variables, supporting the notion that the BCT score has more prognostic power than other prognostic models based on clinical variables alone, and showing that the BCT score provides additional prognostic information with respect to distant metastasis.

Subgroup analysis (according to age, tumor size, histologic grade, pathologic stage and pN status) of patients in the validation cohort treated with hormone therapy alone showed that the BCT score is a significant predictor of distant metastasis in patients aged ≥50 years, and in patients with histologic grade 2 and pN0 status. A limitation of the subgroup analyses is that some of the subgroups contained a small number of patients. The prognostic performance of the BCT score in these subgroups requires assessment in further studies that include larger numbers of patients.

Patients with HR- breast cancer most often experience recurrence within the first 5 years after diagnosis or surgery; the rate of late recurrence is low^39^. By contrast, patients with LN−, HR+ breast cancer remain at high risk for recurrence beyond the first 5 years^4^,^5^. Therefore, it is important to identify late recurrence events in HR+ breast cancer patients. However, reports suggest that the prognostic accuracy of currently available assays may diminish over time, particularly beyond 5 years from diagnosis or primary treatment^40^,^41^. A recent study showed that, while the IHC4 and Oncotype DX 21-RS assays were strong prognostic factors for early recurrence (0–5 years), they did not have a significant prognostic ability to predict late distant recurrence (5–10 years)^42^. However, another recent study reported that ER transcript levels in Oncotype DX 21-RS predict late recurrence in patients with ER+/HER2−43. Importantly, we showed that the BCT score stratified patients into low-risk and high-risk groups after 0–5 years and beyond 5 years, supporting the prognostic value of the BCT score both for early and late recurrence risk in pN0-N1, HR+ breast cancer patients. This may be clinically important in terms of the decision whether to extend adjuvant hormone therapy or initiate adjuvant chemotherapy in patients with a high risk of recurrence beyond 5 years.

Notably, the results also demonstrated that the BCT score can stratify chemotherapy-treated patients into high- and low-risk groups. In this case, the high-risk group had a considerable rate of distant metastasis (21.8%) even after chemotherapy, meaning that extended or more intensive adjuvant treatments may be considered. This suggests that the BCT score may help to identify patients that should be treated with more aggressive adjuvant therapies or novel treatment strategies.

However, the present study has some limitations. Although the prognostic genes used were identified from microarray datasets derived from breast cancer patients in western countries, the prognostic model was developed and validated in Asian cohorts. Additional large validation studies in other populations are needed to further determine the prognostic performance of the BCT score and to compare its prognostic value with that of other prognostic models based on available commercial multigene assays.

In summary, we developed a new molecular prognostic model, called the BCT score, based on expression levels of six prognostic genes and two clinical variables. This score was then used to predict the risk of distant metastasis in patients with pN0-N1, HR+/HER2− breast cancer and validated in independent cohorts. The results show that the BCT score has a prognostic value for late distant metastasis. Notably, the prognostic model took into account expression levels of an immune response-related gene. The BCT score developed herein provided better prognostic information about distant metastasis than other prognostic models based on traditional clinicopathological factors. Consequently, the BCT score may help inform decisions about the need for additional adjuvant therapies in patients with pN0-N1, HR+/HER2− breast cancer.

## Methods

### Ethical statement

This study was approved by the institutional review board (IRB) of the Samsung Medical Center (SMC) (Seoul, Korea) and the Asan Medical Center (AMC) (Seoul, Korea) and performed in accordance with the Declaration of Helsinki. Because the study was retrospective in nature, the requirement for informed consent was waived. Patient information was anonymized and de-identified prior to analysis.

### Patients and tumor samples

A total of 2,133 FFPE tumor samples taken from patients with breast cancer who underwent curative resection of the primary tumor at the SMC or AMC were screened, and clinical information and survival data were collected. Clinical information included age, menopausal status, tumor size, LN status, pathologic stage, histologic grade, use of hormone therapy or adjuvant chemotherapy, and molecular subtype according to ER, PR, and HER2 status (based on IHC or fluorescence *in situ* hybridization [FISH]). ER and PR status by IHC was acquired from the pathological report. The staining was scored using the semi-quantitative Allred score (AS) with a maximum score of 8, and AS >2 was considered as positive^44^. HER2 positivity was defined as described previously^45^. Samples lacking clinical information or tissue blocks were excluded. Finally, we included 973 eligible FFPE tumor samples, i.e., samples from patients with pN0-N1, HR+/HER2− breast cancer with follow up clinical information for more than 10 years after primary surgery. We evaluated the quality of tumor samples and excluded those in which more than 50% of the area was infiltrated by inflammatory cells or showed interstitial fibrosis or fat. We also excluded samples in which the duct and lobule tissue represented less than 30% of the total sample area. RNA was isolated from tumor tissues meeting the above sample quality requirements, and those with an insufficient RNA yield or poor quality RNA were also excluded. Additionally, we checked ER, PR, and HER2 status by real-time qRT-PCR to avoid including other molecular subtypes of breast cancer. Based on qRT-PCR results, samples not considered HR+/HER2− were excluded. Therefore, 906 patients with evaluable samples were included in the study. The detailed inclusion/exclusion criteria are described in Supplementary Fig. S4. Finally, the discovery cohort used to develop the prognostic model comprised 174 patients with pN0-N1, HR+/HER2− breast cancer that were treated with adjuvant hormone therapy alone. Two hundred and twenty-two patients treated with adjuvant hormone therapy alone and 510 patients treated with adjuvant hormone therapy plus chemotherapy were included in the validation cohort.

### RNA extraction and gene expression analysis by qRT-PCR

Total RNA was isolated from the FFPE samples using a Tissue Preparation System device (Siemens AG, Munich, Germany), and qRT-PCR was performed using a QuantiFast Multiplex RT-PCR Kit (Qiagen, Hilden, Germany) and a LightCycler 480 system (Roche Applied Science, Mannheim, Germany). The reaction mixture was dispensed in 384-well plates by an automated dispenser (STARlet; Hamilton Robotics, Reno, NV, USA). qRT-PCR in the validation cohort was conducted by adding a cDNA pre-amplification step to the qPCR protocol. The qRT-PCR results were expressed as quantification cycle (Cq) values.

The Cq value for each gene was reported as the relative expression value normalized against the expression levels of three reference genes (*CTBP1, CUL1*, and *UBQLN1*), the expression of which is highly stable in FFPE tissues^46^. The relative expression value for each gene was calculated based on the difference between the average Cq value for the three reference genes and the target Cq value for each sample as follows:


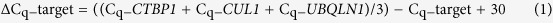


Details of quality assessment for qRT-PCR results from FFPE tissues are presented in Supplementary Methods and Supplementary Fig. S5.

### Identification and selection of prognostic genes

A detailed description of the selection of the six prognostic genes is shown in the Supplementary Fig. S6. We initially selected the 16 genes from the 384 candidate genes identified in our previous study using public gene expression microarray data^28^. Next, the expression of the 16 candidate genes in FFPE tissues from patients with breast cancer was measured by qRT-PCR, and Cox’s proportional hazard analysis was performed to evaluate the association between the expression level of each gene and DMFS. Finally, the expression levels of six prognostic genes, *UBE2C, TOP2A, RRM2, FOXM1, MKI67*, and *BTN3A2*, which are either significant or marginally significant for DMFS in LN−, HR+/HER2− breast cancer, were selected to develop a novel prognostic algorithm to predict the risk of distant metastasis in HR+/HER2− early breast cancer (Table 4). Univariate analysis revealed that expression levels of five proliferation-related genes (*UBE2C, TOP2A, RRM2, FOXM1*, and *MKI67*) were positively correlated with the risk of distant metastasis, whereas high expression of the immune response-related gene, *BTN3A2*, was marginally significant for a decreased risk of distant metastasis in patients with LN−, HR+/HER2− breast cancer (data not shown).

### Development of a prognostic model using clinical and molecular data

We performed univariate and multivariate analysis of the clinicopathological characteristics of the discovery cohort using Cox’s proportional hazards regression model and found that two clinical variables (pN status and tumor size) were independent negative prognostic factors for distant metastasis in pN0-N1, HR+/HER2− breast cancer patients (Supplementary Table S2). Therefore, clinical variables including primary tumor size (cm) and pN status (pN0-N1), in combination with relative expression values for the six prognostic genes normalized according to the average expression level of three reference genes (*CTBP1, CUL1*, and *UBQLN1*), were used to calculate a numerical BCT score, which is a molecular predictor of distant metastasis within 10 years. Missing Cq values were assigned as 45 (the Cq value of the maximum number of cycles used) as described previously^17^,^47^. The coefficient values for each variable were obtained from the Cox’s proportional hazard model, and the unscaled BCT score was defined as a linear combination of the coefficients to predict distant metastasis as follows:


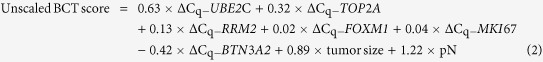


The unscaled BCT score was then re-scaled from 0 to 10 as follows:


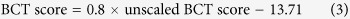


If the calculated BCT score was less than 0, then its value was set as 0, and if the BCT score was more than 10 then the final score was set at 10. Higher BCT scores indicate a higher risk of distant metastasis.

The cutoff BCT score used to distinguish between patients at low and high risk for distant metastasis was set to 4, which maximized the sum of sensitivity and specificity. A patient was assigned to the ‘high-risk’ group if the BCT score of the sample was ≥4. Otherwise, the patient was assigned to the ‘low-risk’ group.

### Validation of the prognostic model

An independent cohort was used to validate the prognostic value of the BCT score. All measurements and analyses were performed at an independent laboratory and were based on procedures predefined in the discovery study. As in the discovery study, the BCT scores were calculated based on the PCR results for six prognostic genes, tumor size, and pN. The primary endpoint for statistical analysis was DMFS, defined as the time from the date of primary surgery to the detection of any distant metastases.

### Comparison of the BCT score with prognostic models based on clinical variables

The prognostic performance of the BCT score was compared with three other prognostic models based on clinical variables: the NPI score^7^,^8^ and two web-based prediction tools, SNAP (www.CancerMath.net)^9^ and PREDICT (www.predict.nhs.uk)^10^,^11^. The NPI score was calculated as follows: 0.2 × tumor size (cm) + tumor grade + nodal status. The NPI prognostic value was calculated for each of the samples, which were then classified into four NPI prognostic groups as follows: excellent, 2–2.4; good, 2.4–3.4; moderate, 3.4–5.4; and poor, >5.4. In SNAP, survival rates are calculated using age, tumor size, LN status, ER/PR/HER2 status, tumor grade, and histologic type. Similarly, PREDICT is a breast cancer prognostication and treatment benefit tool based on prognostic factors such as age, tumor size, tumor grade, number of positive LNs, and ER/HER2/Ki-67 status. The Harrell’s C-index was also calculated to estimate the capacity of each prognostic model to predict the risk of distant metastasis and to compare prognostic performance^48^.

### Statistical analysis

Univariate and multivariate analyses using Cox’s proportional hazard regression models were used to identify genes and clinicopathological variables associated with patient survival. All hazard ratios were reported with 95% CIs. The probability of DMFS was estimated using the Kaplan-Meier method, and the log-rank test was used to assess statistical differences in the survival rates between groups. Differences were considered statistically significant at *P* < 0.05. All statistical analyses were performed using R 3.2.0 (http://r-project.org).

## Additional Information

**How to cite this article:** Gong, G. *et al*. A new molecular prognostic score for predicting the risk of distant metastasis in patients with HR+/HER2− early breast cancer. *Sci. Rep.* **7**, 45554; doi: 10.1038/srep45554 (2017).

**Publisher's note:** Springer Nature remains neutral with regard to jurisdictional claims in published maps and institutional affiliations.

## Supplementary Material

Supplementary Information

## Figures and Tables

**Table 1:** Characteristics of the patients in the discovery and validation cohorts.

	Discovery cohort		Validation cohort				*P value between A and B*	*P value between B and C*
	Hormone therapy alone (A) (*n* = 174)		Hormone therapy alone (B) (*n* = 222)		Hormone therapy plus chemotherapy (C) (*n* = 510)			
	No. of patients	%	No. of patients	%	No. of patients	%		
DMFS rate at 10 years (%)	92.0% (87.9%–96.3%)		92.2% (88.0%–96.6%)		84.7% (81.4%–88.2%)			
Median age (range), years	53.8 (24.3–80.5)		50.0 (29.0–80.0)		46.0 (25.2–67.7)		**<0.002**^**c**^	**<0.013**^**c**^
Age, years							0.052^a^	**<0.001**^**a**^
<50	66	37.9%	107	48.2%	352	69.0%		
≥50	108	62.1%	115	51.8%	158	31.0%		
Menopausal status							**<0.002**^**a**^	**0.004**^**a**^
Pre	65	37.4%	115	51.8%	204	40.0%		
Post	89	51.1%	77	34.7%	75	14.7%		
NA	20	11.5%	30	13.5%	231	45.3%		
pN							0.520^a^	**<0.001**^**a**^
0	163	93.7%	203	91.4%	322	63.1%		
1	11	6.3%	19	8.6%	188	36.9%		
Tumor size (cm)							0.693^a^	**<0.001**^**b**^
≤2	141	81.0%	184	82.9%	252	49.4%		
2–5	33	19.0%	38	17.1%	251	49.2%		
>5	0	0.0%	0	0.0%	7	1.4%		
Pathologic stage							0.624^a^	**<0.001**^**a**^
IA	136	78.2%	177	79.7%	153	30.0%		
IIA	31	17.8%	33	14.9%	258	50.6%		
IIB	7	4.0%	12	5.4%	99	19.4%		
Histologic grade							**0.002**^**a**^	0.203^a^
1	53	30.5%	36	16.2%	80	15.7%		
2	103	59.2%	148	66.7%	313	61.4%		
3	18	10.3%	38	17.1%	117	22.9%		
NPI							0.257^a^	**<0.001**^**a**^
1	130	74.7%	154	69.4%	156	30.6%		
2	36	20.7%	49	22.1%	211	41.4%		
3	8	4.6%	19	8.5%	143	28.0%		

Abbreviations: DMFS, distant metastasis-free survival; NA, not available; No., number; NPI, Nottingham Prognostic Index; pN, pathological nodal status.

^a^Chi-square test; ^b^Fisher’s exact test; ^c^Student’s t-test. *P* values < 0.05 are marked in bold.

**Table 2:** Multivariate analysis of the BCT score and the clinicopathological parameters for DMFS in pN0-N1, HR+/HER2− breast cancer patients treated with hormone therapy alone.

	Discovery cohort			Validation cohort		
	Hazard ratio	95% CI	*P* value	Hazard ratio	95% CI	*P* value
BCT score	4.86	(1.87–12.68)	**0.001**	1.59	(1.12–2.25)	**0.009**
Age at surgery	1.04	(0.98–1.11)	0.158	1.02	(0.96–1.09)	0.460
Tumor size	0.54	(0.14–2.11)	0.374	1.23	(0.60–2.50)	0.566
No. of LN metastasis	0.89	(0.26–3.03)	0.858	0.21	(0.03–1.57)	0.129
Histologic grade	1.06	(0.30–3.76)	0.931	0.86	(0.28–2.68)	0.800
ER (IHC)	1.04	(0.52–2.06)	0.918	1.15	(0.74–1.78)	0.545
PR (IHC)	1.04	(0.77–1.38)	0.816	0.94	(0.78–1.13)	0.511

Abbreviations: CI, confidence interval; ER, estrogen receptor; IHC, immunohistochemistry; LN, lymph node; No, number; PR, progesterone receptor. Hazard ratios with *P* values < 0.05 are marked in bold.

**Table 3:** Multivariate analysis of the ability of the BCT score and other prognostic models based on traditional clinicopathological parameters to predict DMFS in pN0-N1, HR+/HER2− breast cancer patients treated with hormone therapy alone.

	Discovery cohort			Validation cohort		
	Hazard ratio	95% CI	*P* value	Hazard ratio	95% CI	*P* value
BCT score	4.86	(1.87–12.68)	**0.001**	1.59	(1.12–2.25)	**0.009**
NPI Score	>100	(0.00-Inf)	0.997	>100	(0.00-Inf)	0.998
PREDICT	1.01	(0.87–1.18)	0.869	0.91	(0.77–1.09)	0.318
SNAP	1.06	(0.67–1.66)	0.805	0.96	(0.74–1.24)	0.730

Abbreviations: CI, confidence interval; NPI, Nottingham Prognostic Index; PREDICT (www.predict.nhs.uk); SNAP (www.CancerMath.net). Hazard ratios with *P* values < 0.05 are marked in bold.

**Table 4:** The six prognostic genes upon whose expression the BCT algorithm was based.

Gene group	Gene symbol	Full name	GO terms (biological process)
Proliferation	*UBE2C*	Ubiquitin-conjugating enzyme E2C	Cell division; mitotic cell cycle; mitotic spindle assembly checkpoint
	*TOP2A*	Topoisomerase (DNA) II alpha	DNA topological change; mitotic cell cycle
	*RRM2*	Ribonucleotide reductase M2	G1/S transition of mitotic cell cycle; mitotic cell cycle
	*FOXM1*	Forkhead box M1	G2/M transition of mitotic cell cycle; mitotic cell cycle
	*MKI67*	Marker of proliferation Ki-67	DNA metabolic process; cell proliferation
Immune response	*BTN3A2*	Butyrophilin, subfamily 3, member A2	T cell-mediated immunity; interferon-gamma secretion

Abbreviation: GO, gene ontology.
