# Supplementary material for: A new molecular prognostic score for predicting the risk of distant metastasis in patients with HR+/HER2− early breast cancer
Source: Sci Rep. 2017 Mar 28;7:45554. doi: 10.1038/srep45554 (PMC5368569; doi:10.1038/srep45554)
Supplement: Supplementary Information [file srep45554-s1.pdf]

A new molecular prognostic score for predicting the risk of distant metastasis in patients with HR+/HER2- early breast cancer

Gyungyub Gong[*], Mi Jeong Kwon[*], Jinil Han, Hee Jin Lee, Se Kyung Lee, Jeong Eon Lee, Seon-Heui Lee, Sarah Park, Jong-Sun Choi, Soo Youn Cho, Sei Hyun Ahn, Jong Won Lee, Sang Rae Cho, Youngho Moon, Byung-Ho Nam, Seok Jin Nam, Yoon-La Choi, Young Kee Shin

[*] These authors contributed equally to this study.

Corresponding author: Young Kee Shin (ykeeshin@snu.ac.kr)

Supplementary Methods

RNA extraction and gene expression analysis by qRT-PCR

FFPE tissue-derived RNA degradation can be increased with storage time of FFPE tissue samples and increased RNA degradation can affect the qRT-PCR results [1]. The FFPE tumor samples used in this study were stored at room temperature and their storage time ranged between 8 and 18 years. To assess the effect of storage time of FFPE tissues on qRT-PCR results, we compared the qRT-PCR data from the FFPE tissues stored for different periods of time in our discovery cohort using mean Cq values for six prognostic genes. Relatively higher mean Cq values were observed for FFPE specimens stored for longer than 10 years compared with those stored for less than 10 years (Supplementary Fig. S5A). These results indicated that RNA degradation increases with storage time. However, consistent with a previous study by Cronin *et al*.[1], normalization by three reference genes corrected for this bias (Supplementary Fig. S5B).

References

1 Cronin, M. *et al.* Measurement of gene expression in archival paraffin-embedded tissues: development and performance of a 92-gene reverse transcriptase-polymerase chain reaction assay. *Am J Pathol* **164**, 35-42, doi:S0002-9440(10)63093-3

Supplementary Tables

Supplementary Table S1. BCT score hazard ratios according to age, tumor size, histologic grade, pathologic stage and pN status in the discovery and validation cohorts

BCT score (continuous variable)												
	Discovery cohort				Validation cohort							
	Hormone therapy alone (*n* = 174)				Hormone therapy alone (*n* = 222)				Hormone therapy plus chemotherapy (*n* = 510)			
	No	Hazard ratio	95% CI	*P* value	No	Hazard ratio	95% CI	*P* value	No	Hazard ratio	95% CI	*P* value
Total	174	2.89	(1.89-4.43)	**<0.001**	222	1.38	(1.12-1.70)	**0.003**	510	1.37	(1.22-1.55)	**<0.001**
Age, years												
<50	66	5.06	(1.50-17.07)	**0.009**	107	2.27	(0.90-5.70)	0.082	352	1.44	(1.23-1.68)	**<0.001**
≥50	108	2.35	(1.46-3.78)	**<0.001**	115	1.26	(1.00-1.59)	**0.049**	158	1.28	(1.06-1.55)	**0.011**
Tumor size, cm												
≤2	141	4.16	(1.77-9.76)	**0.001**	184	1.24	(0.91-1.70)	0.175	252	1.40	(1.14-1.73)	**0.001**
>2	33	5.01	(1.56-16.09)	**0.007**	38	1.43	(0.92-2.22)	0.111	258	1.40	(1.19-1.65)	**<0.001**
Histologic grade												
1	53	3.70	(1.28-10.68)	**0.016**	36	40.52	(0.00-1260562.00)	0.483	80	1.42	(1.06-1.90)	**0.017**
2	103	2.68	(1.52-4.71)	**0.001**	148	1.51	(1.10-2.07)	**0.011**	313	1.48	(1.25-1.75)	**<0.001**
3	18	3.47	(1.13-10.64)	**0.029**	38	1.25	(0.81-1.92)	0.314	117	1.14	(0.90-1.45)	0.278
Pathologic stage												
1	136	3.77	(1.44-9.86)	**0.007**	177	1.32	(0.94-1.85)	0.111	153	1.36	(0.94-1.97)	0.107
2	38	2.94	(1.34-6.48)	**0.007**	45	1.30	(0.93-1.83)	0.129	357	1.34	(1.18-1.54)	**<0.001**
pN status												
pN0	163	2.94	(1.69-5.11)	**<0.001**	203	1.49	(1.18-1.88)	**0.001**	322	1.31	(1.10-1.56)	**0.003**
pN1	11	1.59	(0.72-3.49)	0.247	19	1.21	(0.56-2.60)	0.627	188	1.41	(1.17-1.69)	**<0.001**
BCT score (categorical variable)												
	No	Hazard ratio	95% CI	*P* value	No	Hazard ratio	95% CI	*P* value	No	Hazard ratio	95% CI	*P* value
Total	174	16.88	(5.18-55.01)	**<0.001**	222	8.57	(2.80-26.25)	**<0.001**	510	2.72	(1.63-4.54)	**<0.001**
Age, years												
<50	66				107	5.68	(0.51-63.08)	0.157	352	2.49	(1.36-4.57)	**0.003**
≥50	108	10.03	(2.82-35.71)	**<0.001**	115	7.59	(1.96-29.40)	**0.003**	158	3.29	(1.22-8.87)	**0.019**
Tumor size, cm												
≤2	141	23.22	(5.10-105.90)	**<0.001**	184	4.77	(0.92-24.59)	0.062	252	2.45	(1.21-4.96)	**0.013**
>2	33	NA			38	NA			258	3.79	(1.48-9.70)	**0.006**
Histologic grade												
1	53	46.40	(3.87-555.90)	**0.002**	36	NA			80	6.00	(1.09-32.94)	**0.039**
2	103	14.48	(2.81-74.71)	**0.001**	148	8.78	(2.35-32.77)	**0.001**	313	2.87	(1.50-5.47)	**0.001**
3	18	12.51	(1.04-151.20)	**0.047**	38	NA			117	1.15	(0.42-3.11)	0.785
Pathologic stage												
1	136	28.63	(4.70-174.30)	**0.000**	177	6.17	(1.20-31.85)	**0.030**	153	2.62	(0.86-8.00)	0.092
2	38	6.60	(0.81-53.71)	0.078	45	NA			357	2.40	(1.29-4.46)	**0.006**
pN status												
pN0	163	15.43	(3.68-64.69)	**<0.001**	203	10.90	(3.45-34.41)	**<0.001**	322	2.89	(1.34-6.21)	**0.007**
pN1	11	3.70	(0.41-33.81)	0.246	19	NA			188	1.97	(0.98-3.95)	0.056

Abbreviations: CI, confidence interval; NA, not available; No., number of patients; pN, pathological nodal status. Hazard ratios with *P* values < 0.05 are marked in bold.

Supplementary Tables S2. Univariate and multivariate analysis of clinical variables for DMFS in HR+/HER2- early breast cancer patients in the discovery cohort

		Univariate analysis			Multivariate analysis		
		Hazard ratio	95% CI	*P* value	Hazard ratio	95% CI	*P* value
Age		1.05	(1.00–1.10)	0.058	1.03	(0.98–1.08)	0.234
Tumor size		4.31	(2.11–8.81)	**<0.001**	3.7	(1.18–11.64)	**0.025**
pN	0	1			1		
	1	11.78	(3.82–36.30)	**<0.001**	13.86	(2.20–87.56)	**0.005**
Pathologic stage	I	1			1		
	II	6.51	(2.12–19.93)	**0.001**	1.38	(0.16–12.19)	0.773
Histologic grade	1	1			1		
	2	1.12	(0.29–4.34)	0.867	3	(0.37–24.38)	0.303
	3	2.94	(0.59–14.59)	0.186	18.9	(0.62–578.68)	0.092
NPI	1	1			1		
	2	6.6	(1.93–22.54)	**0.003**	0.55	(0.06–4.92)	0.593
	3	9.46	(1.73–51.78)	**0.01**	0.06	(0.00–3.02)	0.162

Abbreviations: CI, confidence interval; NPI, Nottingham prognostic index; pN, pathologic nodal status. Hazard ratios with *P* values < 0.05 are marked in bold.

Supplementary Figures

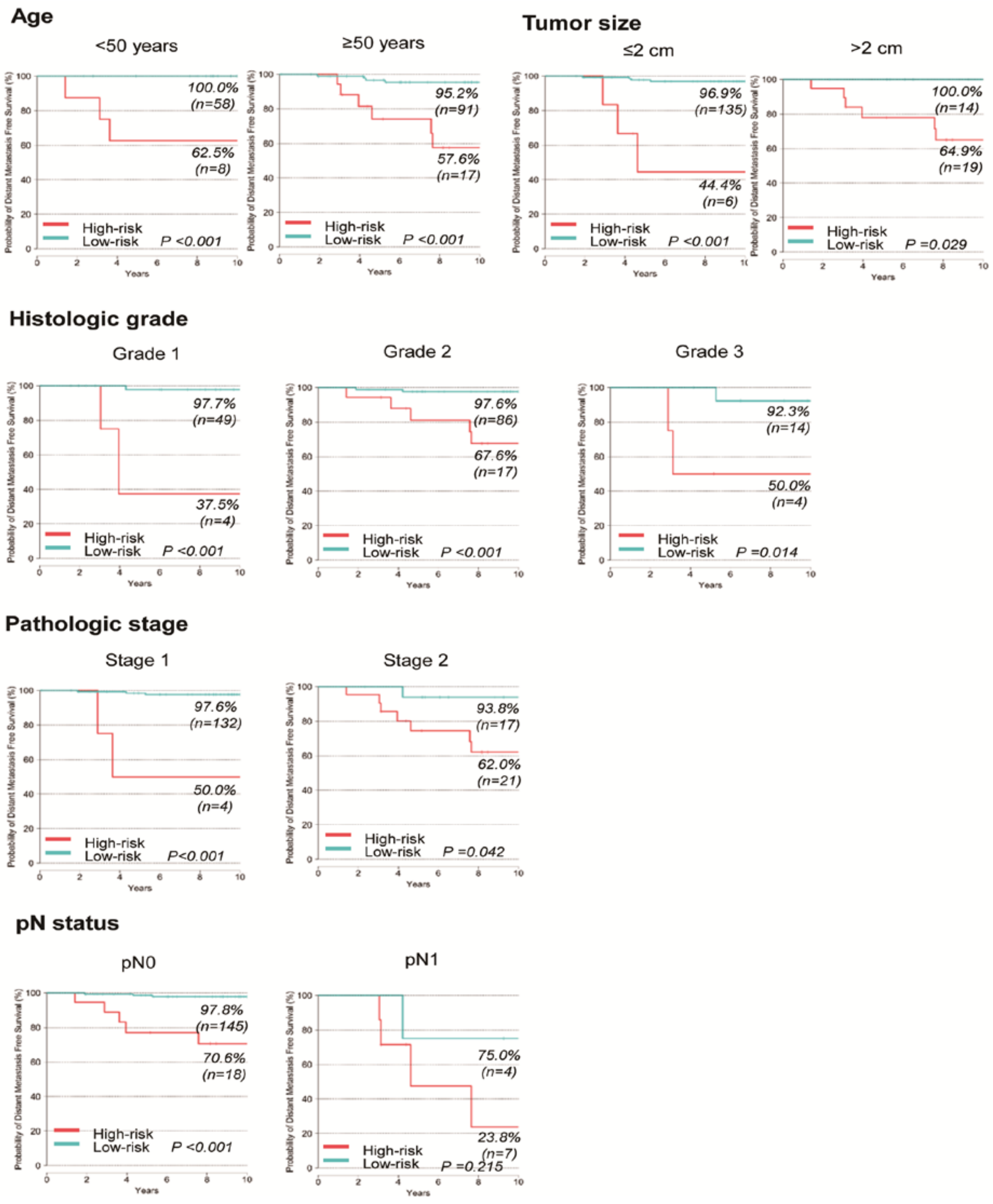

Supplementary Figure S1. Kaplan-Meier analyses in subgroups of the discovery cohort. Patients were subdivided according to age, tumor size, pN status, histologic grade, and pathologic stage. For each subgroup, the results of high- and low-risk groups (defined by the BCT score) are shown.

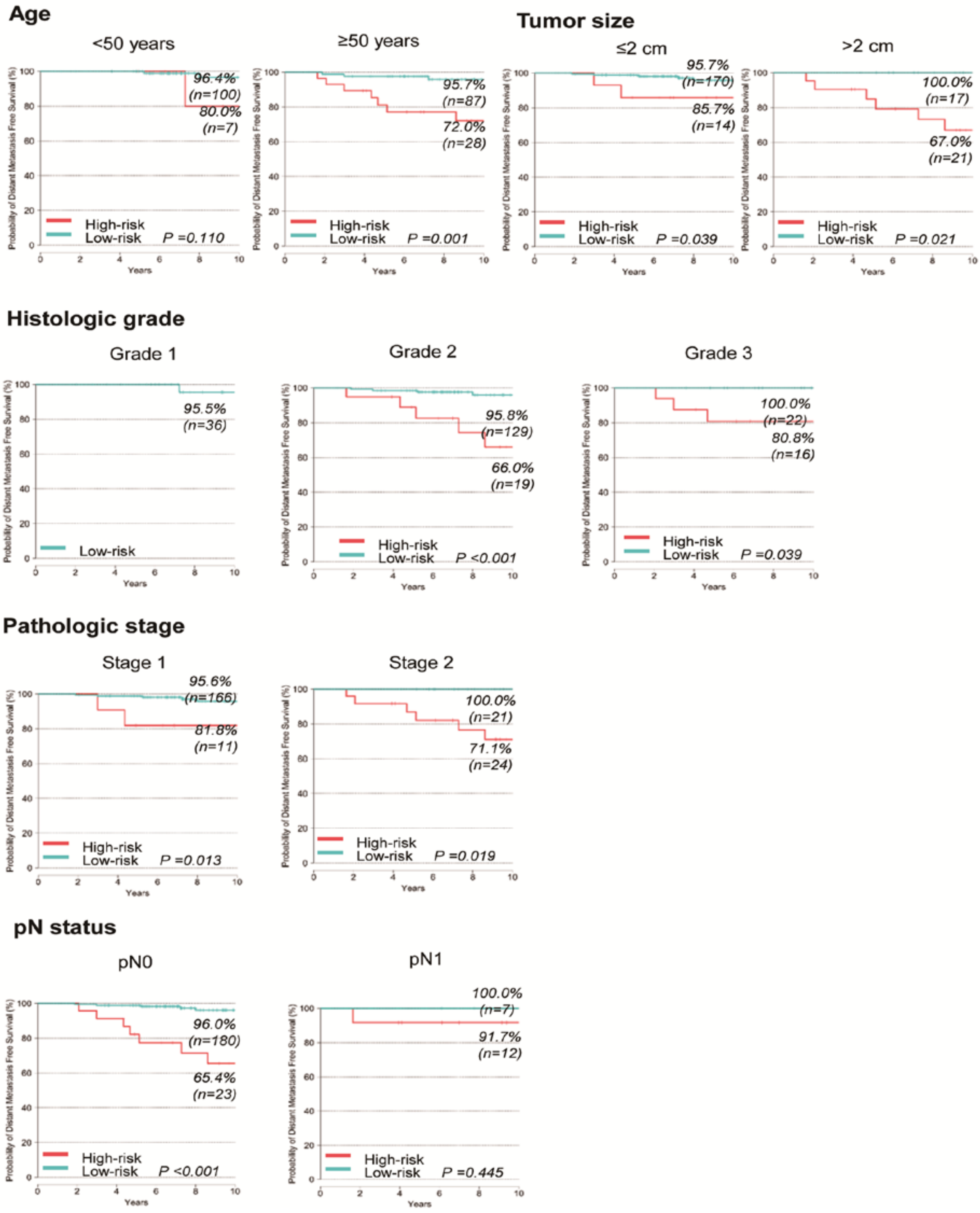

Supplementary Figure S2. Kaplan-Meier analyses in subgroups of the validation cohort treated with hormone therapy alone. Patients were subdivided according to age, tumor size, pN status, histologic grade, and pathologic stage. For each subgroup, the results of high- and low-risk groups (defined by the BCT score) are shown.

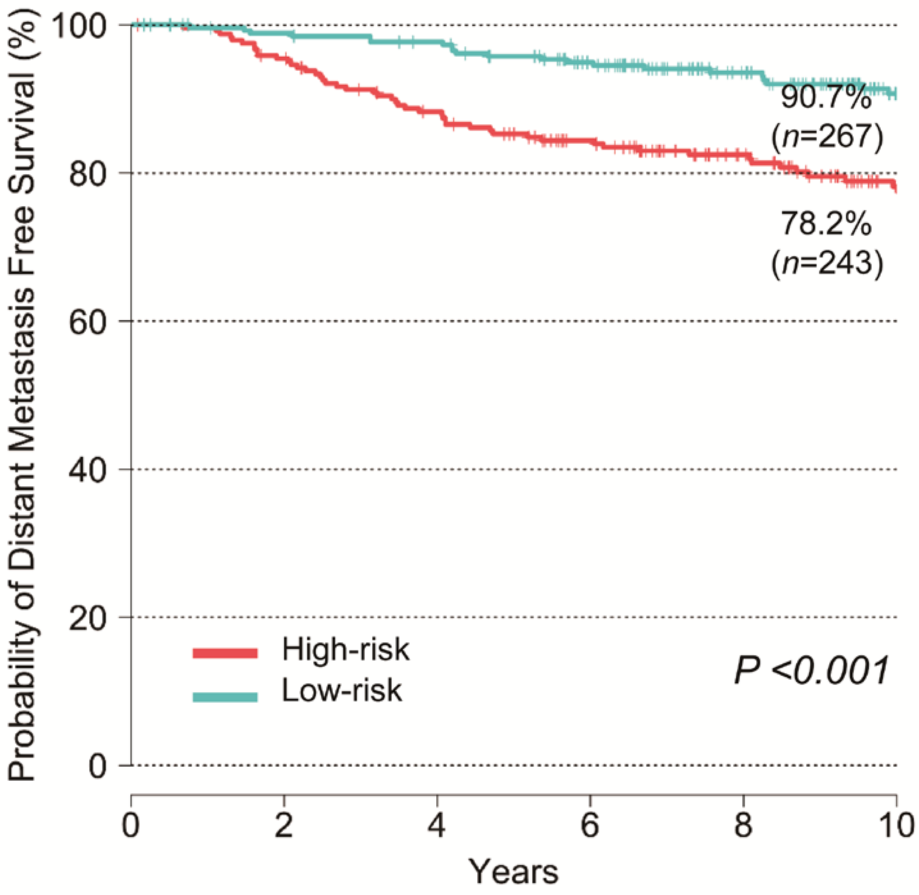

Supplementary Figure S3. Kaplan-Meier plots of distant metastasis in high- and low-risk groups (defined by the BCT score) of chemotherapy-treated patients (n = 510). The cutoff value for the BCT score was 4.

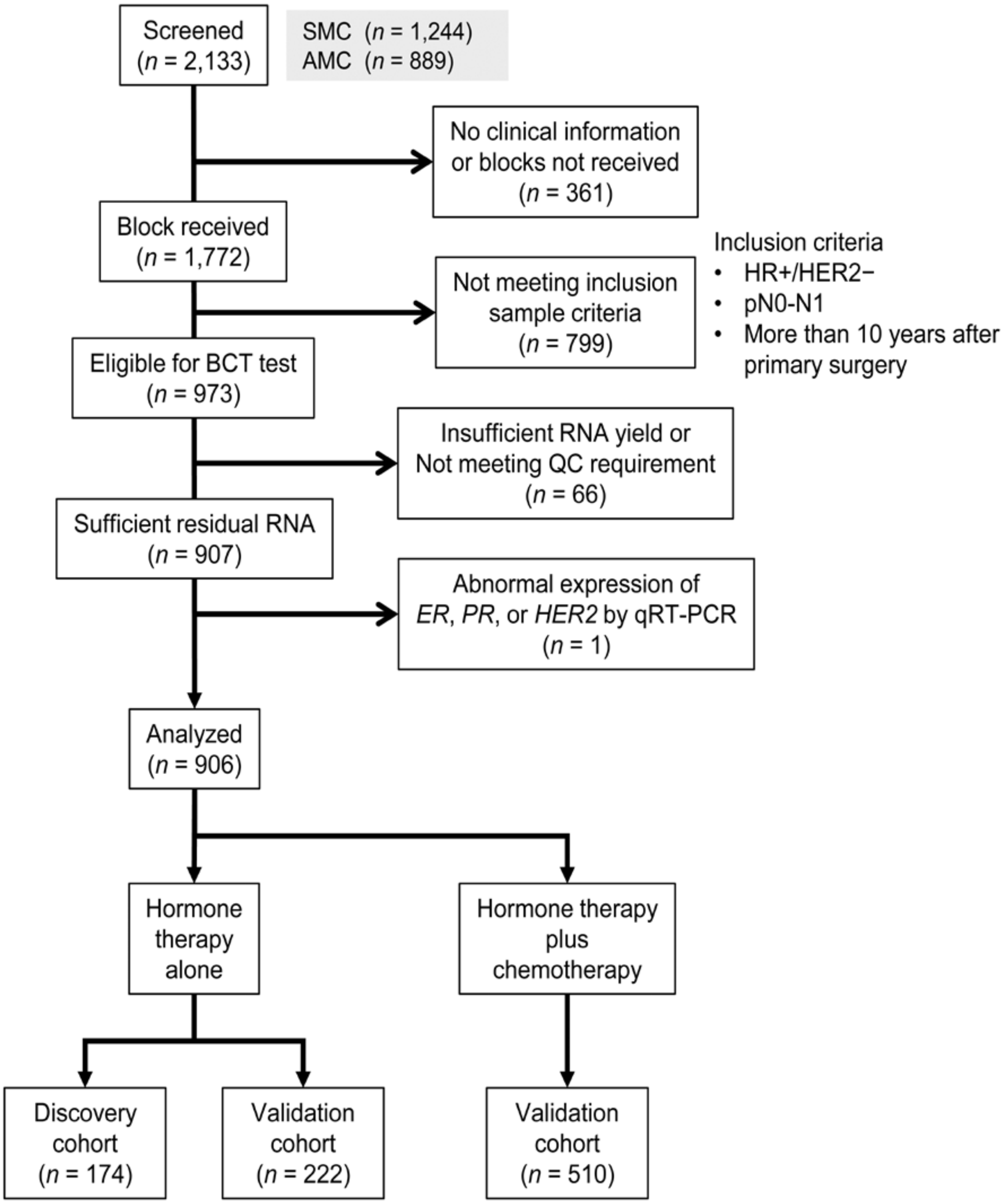

Supplementary Figure S4. Flow diagram of the study. AMC: Asan Medical Center. SMC: Samsung Medical Center. QC: quality control.

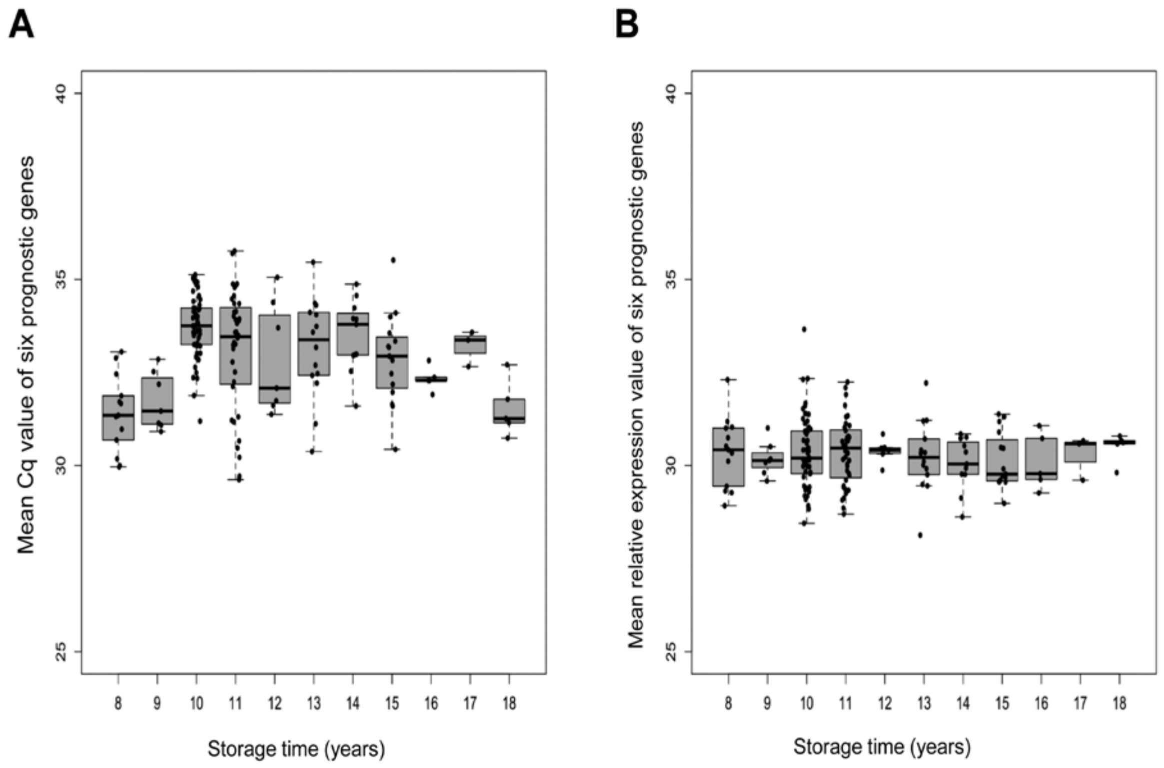

Supplementary Figure S5. Mean Cq values and relative expression values for six prognostic genes in discovery cohort as a function of FFPE tissue storage time. (A) Mean Cq values for six prognostic genes in our discovery cohort (n = 174). (B) Mean expression values after normalization relative to three reference genes (*CTBP1*, *CUL1* and *UBQLN1*) as described in the Methods section. The x-axis represents the storage time of each specimen and y-axis shows the mean Cq values or relative expression values after normalization by three reference genes.

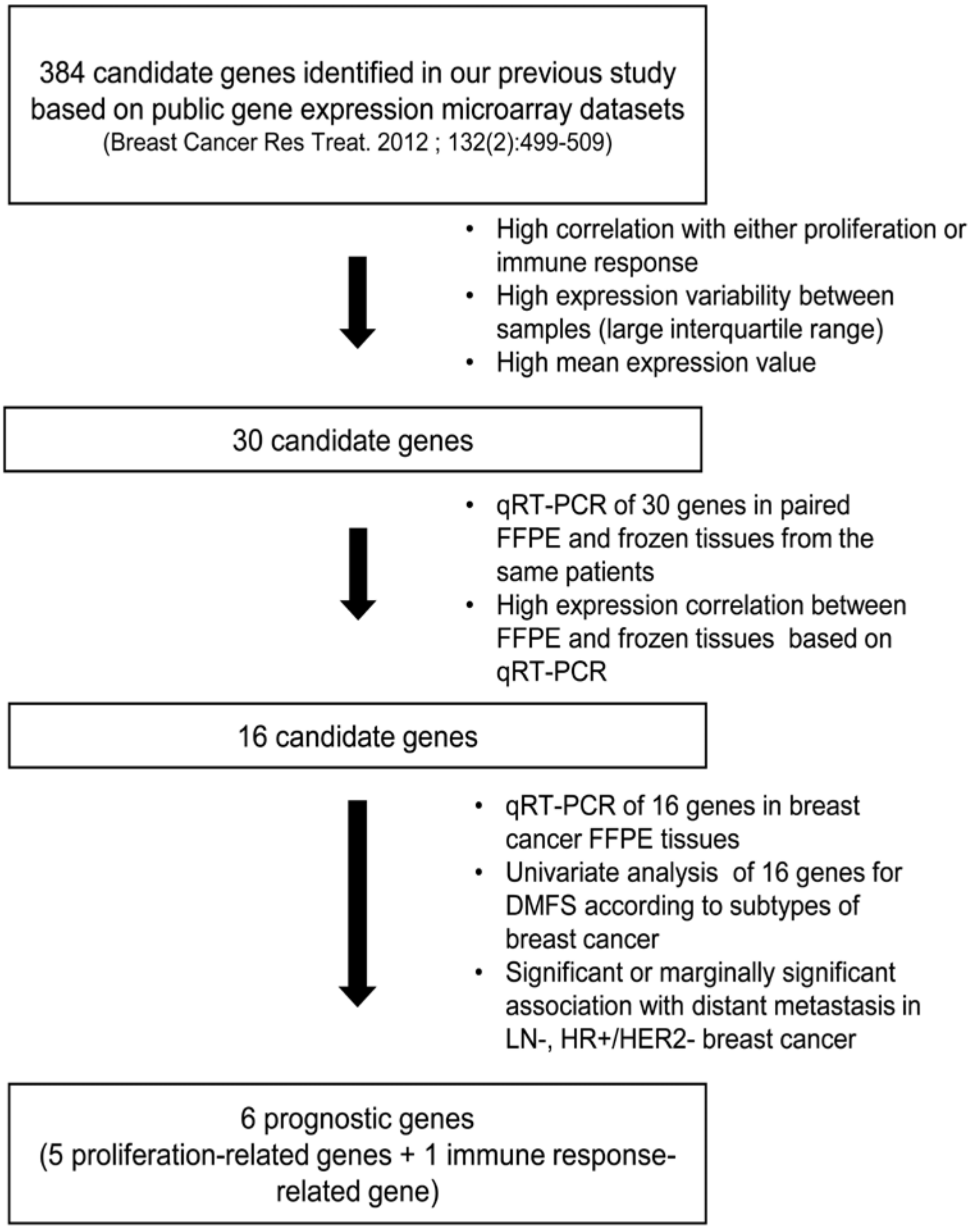

Supplementary Figure S6. Prognostic gene selection process
